# Competition and private R&D investment

**DOI:** 10.1371/journal.pone.0232119

**Published:** 2020-05-27

**Authors:** Thomas Grebel, Lionel Nesta

**Affiliations:** 1 TU Ilmenau, Economics Department, Ilmenau, Germany; 2 Groupe de Recherche en Droit, Economie, Gestion, Université Cote d’Azur, France; 3 SciencesPo, OFCE, Paris, France; 4 SKEMA Business School, Sophia-Antipolis, France; Washington State University, UNITED STATES

## Abstract

We investigate the determinants of the sign of Research and Development reaction functions of rival firms. Using a two-stage *n*-firm Cournot competition game, we show that this sign depends on four types of environments in terms of product rivalry and technology spillovers. We test the predictions of the model on the world’s largest manufacturing corporations. Assuming that firms make R&D investments based on the R&D effort of the representative rival company, we develop a dynamic panel data model that accounts for the endogeneity of the decision of the rival firm. Empirical results thoroughly corroborate the validity of the theoretical model.

## Introduction

A striking outcome of the recent paper by [1] is that the relationship between a firm’s own R&D and that of a product market rival is ambiguous. The slope of the R&D reaction function, whether positive or negative, depends on how the research effort by the rival company affects the profitability of the firm’s own R&D. Our intuition is that when studying R&D reaction functions, one must first determine the context within which any two firms compete in terms of technology spillovers and product market rivalry. This mix determines whether the R&D investments by two companies are strategic complements or substitutes.

The question of the complementarity or substitution of R&D investments in the presence of spillovers by firms is crucial. Due to positive externalities, such investments are typically seen as strategic substitutes [2, 3, 4]. More recently, Strandholm et al. [5] show that the social welfare effects of policies are crucially dependent on the presence of technology spillovers. However, these contributions implicitly assume that firms are rivals on the product market. Instead, imagine that firms sell complementary products. Our intuition is that an increase in a firm’s own R&D may very well encourage its strategic complement to also increase its research efforts. A better understanding of such mechanisms would help to design better R&D policies supporting private research [6].

This article develops a two-stage non-cooperative Cournot model that reconciles the views that technology spillovers may either impede or conversely motivate firm R&D investments. A key assumption of the model is that the goods produced by the two firms are imperfect substitutes [7, 8]. The rationale is straightforward. If firms do business in complementary or independent markets, they do not compete in output. Technology spillovers may then be beneficial or harmless to both companies because they do not reduce a firm’s market size. Conversely, if products are close substitutes, technology spillovers may enter the production function of the rival company. Whether firms reap profits from their research efforts depends on the degree of knowledge spillovers and of product substitution. It is this mix between technology spillovers on the one hand and product market competition on the other that will determine whether R&D investments between any two companies are complements or substitutes.

This article also develops an empirical version of the R&D reaction function and applies it to data on the world’s largest companies. The combination of patent data from the USPTO and financial information from Compustat of 308 companies allows us to determine the degree of technological spillovers and of product substitution for any dyad of firms. Because companies cope with an array of competitors, we assume that firms make *oblivious* R&D investments based on the R&D decision of the *representative* rival company. This assumption allows us to empirically determine the sign of the R&D reaction function. Pre-sample mean panel data models accounting for the endogeneity of the R&D decision by the rival company corroborate the theoretical predictions.

The originality of this article is threefold. First, on the theoretical side, we concentrate exclusively on the sign of the R&D reaction function. By doing so, we show that the sign is fully determined by the degree of technology spillover and product market rivalry. Second, on the empirical side, all contributions treat technology spillovers and/or product market rivalry as determinants of innovation, profitability, or market value. Instead, we consider technology spillovers and product market rivalry as the elements that provide a context within which two rival firms determine their level of R&D efforts. Third, we develop an empirical version of the theoretical R&D reaction function that accounts for the simultaneity of such decisions using the generalized method of moments. Our results are consistent with the theoretical framework, implying that contrary to the usual wisdom, spillovers may spur firm R&D investments.

Section (1) introduces the model. Section (2) investigates the conditions that determine the positive and negative correlations between the firms’ process R&D. Sections (3) and (4) present the empirical protocol and discuss the results. Section (5) concludes.

## 1 The model

The model builds on the contribution by De Bondt Veugelers [7]. We consider *n* firms that produce differentiated goods in quantity *q*_*i*_ with *i* = {1, 2, …, *n*}, with the numeraire good *m*. As in Lin and Saggi [8], who develop a duopoly model along previous work such as by Dixit [9] and Vives [10] substantiating entry barriers and discussing the role of information and competitive advantages, we employ a representative consumer’s utility function associated with the consumption of differentiated goods. The utility function we use takes a quadratic form as in Amir et al. [11]:

$$\begin{array}{c} U\left(q\right)=aq-\frac{1}{2}q\Sigma q+m \end{array}$$ (1)

with *a* as a positive *n*–vector and Σ as a symmetric *n* × *n*–matrix with a diagonal of value 1 and *σ* else. Parameter *σ* represents the degree of substitution between products. Unlike Lin and Saggi [8] and identical to Bondt Veugelers [7], we allow *σ* to be either negative or positive: −1 ≤ *σ* ≤ 1. A positive value for *σ* implies that products are substitutive (i.e., low product differentiation), whereas a negative value entails complementarity between goods. This utility function suggests both a preference for variety—because of its quadratic terms—and a taste for product differentiation—because of the negative effect of *σ* on consumer utility.

The inverse demand function derived from the quadratic utility function from above leads to:

$$\begin{array}{c} p_{i}=a-bq_{i}-\sigma b\sum_{j\neq i}q_{j} \end{array}$$ (2)

with $q_{i}+\sum_{i\neq 1}^{n}q_{i}=Q<a/b$. Note that if *σ* > 0 (resp. *σ* = 1), the products are (resp. perfect) substitutes, implying that firms compete in an oligopoly market. If instead *σ* < 0, products are complementary: an increase in the demand for one product increases the demand for complementary products, leading to an increase in its price. If *σ* = 0, products are entirely unrelated, and firms operate as monopolists in different markets. Hence, an increase in the degree of product differentiation (i.e., a decrease in *σ*), denotes an outward shift of the demand curve for firms.

Firms face constant marginal cost *A*, which can be reduced by means of process R&D *x*_*i*_. As in D’aspremont and Jacquemin [12], firms face externalities in process R&D, depicted by parameter *β* which indicates the spillovers from remaining firms’ process R&D. The marginal cost of production is computed as

$$\begin{array}{c} C_{i}=A-x_{i}-\beta \sum_{j\neq i}x_{j} \end{array}$$ (3)

where 0 < *A* < *a* and *x*_*i*_ + *β*∑_*j*≠*i*_ *x*_*j*_ < *A*. As in De Bondt and Veugelers [7], we assume −1 < *β* < 1. Positive externalities (*β* > 0) imply positive R&D spillovers due to a lack of appropriability. The case for negative externalities (*β* < 0) is admittedly more subtle, but they may stem from factor market imperfections which increase rival firms’ marginal cost. We mainly consider skill-biased technical change, which, by increasing the demand for skilled labor, increase their equilibrium wage for the entire population of firms. Hence, the mathematical continuum of the interval for *β* should not conceal the difference in nature that exists between a positive *β*, which is mainly technological, and a negative *β*, which is mainly pecuniary.

We assume convex costs in process R&D investment, $\gamma x_{i}^{2}/2$, with efficiency parameter *γ* > 0. The profit function reads:

$$\begin{array}{c} \pi_{i}^{q}=[p_{i}-C_{i}]q_{i}-\frac{\gamma }{2}x_{i}^{2} \end{array}$$ (4)

where *p*_*i*_ and *C*_*i*_ are defined by Eqs (2) and (3), respectively.

Altogether, the structure of the game is as follows. In the first stage, firms choose optimal R&D investments. In the second stage, firms decide on optimal production quantity. The firm’s maximization problem is solved by backward induction. Therefore, we first consider the output stage and thereafter the R&D stage.

### Output stage

Firms choose optimal output levels to maximize profits. The first-order condition with respect to *q*_*i*_ reads:

$$\begin{array}{c} a-2bq_{i}-\sigma b\left(Q-q_{i}\right)-C_{i}=0, \end{array}$$ (5)

where $Q=\sum_{i=1}^{n}q_{i}$ denotes total market output. Summing the first-order conditions over all *i* = 1, ‥, *n* gives

$$\begin{array}{c} na-b\left(2+\left(n-1\right)\sigma \right)Q-C^{\Sigma }=0 \end{array}$$

with $C^{\Sigma }=\sum_{i=1}^{n}C_{i}$ and $\partial^{2}\pi_{i}^{q_{i}}/\partial {q_{i}}^{2}=-2>0\;\forall \;b>0$, q* determining a maximum. This leads to total output in equilibrium

$$\begin{array}{c} Q^{*}=\frac{na-C^{\Sigma }}{b(2+(n-1)\sigma )}. \end{array}$$ (6)

Inserting Eq (6) into Eq (5) leads to equilibrium output of the representative firm:

$$\begin{array}{c} q_{i}^{*}=\frac{\left(2-\sigma \right)a-\left(2+\left(n-2\right)\sigma \right)C_{i}+\sigma C_{-i}}{b(\sigma -2)((n-1)\sigma +2)} \end{array}$$ (7)

with *C*_−*i*_ = *C*^Σ^ − *C*_*i*_. Equilibrium profit can then be written as:

$$\begin{array}{c} \pi_{i}^{q*}=a-bq_{i}^{*}-b\sigma \sum_{j\neq i}^{n}q_{j}^{*}-C_{i})q_{i}^{*}-\frac{\gamma }{2}x_{i}^{2} \end{array}$$ (8)

Assuming n = 2 and setting *σ* to unity yields equilibrium output $q_{i}^{*}$ and profit $\pi_{i}^{q*}$, identical to d’Aspremont and Jacquemin [12]. Setting *β* to zero instead yields optimal output $q_{i}^{*}$ and profit $\pi_{i}^{q*}$ identical to Lin and Saggi [8].

### Process R&D stage

The first-order condition in Eq (5) is equivalent to $\left(p_{i}-C_{i}\right)=bq_{i}^{*}$. Hence, the reduced form of the profit function in the process R&D stage is:

$$\begin{array}{c} \pi_{i}^{q*}=b{\left(q_{i}^{*}\right)}^{2}-\frac{\gamma }{2}x_{i}^{2} \end{array}$$ (9)

To obtain the optimal level of process R&D, the respective first-order condition at this stage reads:

$$\begin{array}{c} 2bq_{i}^{*}\frac{\partial q_{i}^{*}}{\partial x_{i}}-\gamma x_{i}=0 \end{array}$$

From Eq (3), we deduce that a one unit change in *x*_*i*_ changes marginal costs *C*_*i*_ by minus one unit, i. e. ∂*C*_*i*_/∂*x*_*i*_ = −1. As the symmetry assumption of firms also holds at the R&D stage, we can state that $\partial q_{i}^{*}/\partial x_{i}=\kappa$, for all *i* = 1, …, *n*. Using this information together witch Eq (7), we can derive

$$\kappa =-\frac{\partial q_{i}^{*}}{\partial C_{i}}-\beta \left(n-1\right)\frac{\partial q_{i}^{*}}{\partial C_{j}},\;\text{for}\;j\neq i$$ (10)

$$=\frac{2+(n-2)\sigma }{b(2-\sigma )(2+(n-1)\sigma )}-\beta \left(n-1\right)\frac{\sigma }{b(2-\sigma )(2+(n-1)\sigma )}$$ (11)

$$=\frac{2-\sigma +(n-1)\sigma (1-\beta )}{b(2-\sigma )(2+(n-1)\sigma )}.$$ (12)

Summing over all first-order conditions at R&D stage yields the first-order condition:

$$\begin{array}{c} 2bQ^{*}\kappa =\gamma nx^{*} \end{array}$$ (13)

where x* denotes the equilibrium value of firm-level R&D. Inserting the marginal cost function *C*_*i*_ in Eq (3) into Eq (6), we obtain

$$\begin{array}{c} Q^{*}=\frac{n\left(a-A\right)+n\left(1+\left(n-1\right)\beta \right)x^{*}}{b(2+(n-1)\sigma )}. \end{array}$$

Now, we can calculate firm-level R&D, plugging Q* and *κ* into Eq (13). This renders equilibrium R&D

$$\begin{array}{c} x^{*}=\frac{2(2-\sigma +(n-1)\sigma (1-\beta ))\left(a-A\right)}{\gamma b\left(2-\sigma \right){\left(2+\left(n-1\right)\sigma \right)}^{2}-2(2-\sigma +(n-1)\sigma (1-\beta ))\left(1+\left(n-1\right)\beta \right))} \end{array}$$ (14)

In the duopoly case (*n* = 2), this reduces to

$$\begin{array}{c} x^{*}=\frac{\left(a-A)(2-\beta \sigma \right)}{\frac{b}{2}\gamma \left(2-\sigma \right){\left(2+\sigma \right)}^{2}-\left(2-\beta \sigma \right)\left(1+\beta \right)}. \end{array}$$ (15)

Note that this result is in line with the model from d’Aspremont Jacquemin [12]. By setting *σ* = 1, optimal process R&D investment (x*) corresponds to the non-cooperative version of their model.

At this stage, in previous contributions such as d’Aspremont Jacquemin [12], Bondt Veugelers [7], Lin and Saggi [8] and Strandholm et al. [5], a discussion of the welfare effects of rivalry in R&D investments and on product markets follows. We provide such an analysis of the welfare effect of product rivalry and technology spillovers in Appendix (A). However, our contention is that product rivalry and the presence of technology spillovers modify the firms’ incentives to invest in research activities. Next Section focuses on this particular issue.

## 2 R&D reaction functions in the *β*-*σ* space

As we focus on strategic R&D investment behavior, we now investigate the reaction function *R*_*i*_(*x*_*j*_) with varying values of *σ* and *β* while assuming symmetric reactions with uniform parameters *a*, *A*, *b*, *γ*, *β* and *σ*. This implies that firm *i* responds to the R&D investments of the remaining (*n*–1) firms. In the following steps, we derive the R&D reaction function *R*_*i*_(*x*_*j*_): inserting q* from Eq (7) into Eq (9), replacing *C*_*i*_ = *A* − *x*_*i*_ − *β*(*n* − 1)*x*_*j*_, and *C*_−*i*_ = (*n* − 1)*A* − (*n* − 1)*βx*_*i*_ − (1 − (*n* − 2))∑_*j*≠*i*_ *x*_*j*_, yields the full form of the R&D-stage profit funtion:

$$\begin{array}{c} \pi^{q^{*}}\left(x_{i},x_{j}\right)=\frac{(\left(\sigma -2\right)\left(a-A\right)+x_{i}\left(\sigma \left(\beta \left(n-1\right)-n+2\right)-2\right)+\left(n-1\right)\left(\sigma -2\beta \right)x_{j}){}^{2}}{b{\left(\sigma -2\right)}^{2}{\left(\left(n-1\right)\sigma +2\right)}^{2}}-\frac{\gamma x_{i}^{2}}{2} \end{array}$$

Setting the first derivative of this profit function to zero and solving for *x*_*i*_ leads to the following reaction function:

$$\begin{array}{c} R_{i}\left(x_{j}\right):x_{i}=\Psi \left(2-\sigma \right)\left(a-A\right)-\Psi \left(n-1\right)\left(\sigma -2\beta \right)x_{j} \end{array}$$ (16)

with $\Psi =\frac{2(2-\sigma +(\beta -(\beta -1)n-2))}{b^{2}\gamma {\left(\sigma -2\right)}^{2}{\left(\left(n-1\right)\sigma +2\right)}^{2}-2{\left(\sigma \left(\beta \left(1-n\right)+n-2\right)+2\right)}^{2}}$. The equation is linear in *x*_*j*_ and describes the best response of firm *i* to the average optimal R&D investments of the representative competitor (note, in equilibrium: *x*_*j*_ = x*).

Equivalently, we assume, below in our empirical exercise, that firms do not react in their R&D investment decisions to a single competitor but rather to a representative competitor. To align the model to our empirical design, we also reduce our model to the case *n* = 2. The corresponding R&D reaction function thus reads:

$$\begin{array}{c} R_{i}\left(x_{j}\right):x_{i}=\frac{-2\left(a-A\right)\left(2-\sigma \right)\left(2-\beta \sigma \right)/b(4-\sigma^{2}{)}^{2}}{\frac{2{\left(\beta \sigma -2\right)}^{2}}{b(4-\sigma^{2}{)}^{2}}-\gamma }+\frac{2\left(2\beta -\sigma \right)\left(\beta \sigma -2\right)/b(4-\sigma^{2}{)}^{2}}{\frac{2{\left(\beta \sigma -2\right)}^{2}}{b(4-\sigma^{2}{)}^{2}}-\gamma }x_{j} \end{array}$$ (17)

with *i*, *j* = 1, 2 and *i* ≤ *j*. The denominators of the two summands in Eq (17) reflect the second-order condition in the R&D stage and must be negative. Observe that Eq (17) is a linear function of the form *x*_*i*_ = *α* + *ωx*_*j*_. Computing *dx*_*i*_/*dx*_*j*_ yields:

$$\begin{array}{c} \frac{dx_{i}}{dx_{j}}=\omega =\frac{-2\left(a-A\right)\left(2-\beta \sigma \right)\left(2\beta -\sigma \right)/b(4-\sigma^{2}{)}^{2}}{\frac{2{\left(2-\beta \sigma \right)}^{2}}{b(4-\sigma^{2}{)}^{2}}-\gamma }. \end{array}$$ (18)

Eq (18) clearly shows that the sign of the effect of firm *j*’s investment in process R&D on firm *i*’s own investment in process R&D, hence parameter *β*, depends on the joint conditions of product substitution *σ* and research spillovers *β*, ceteris paribus.

**Fig 1:**
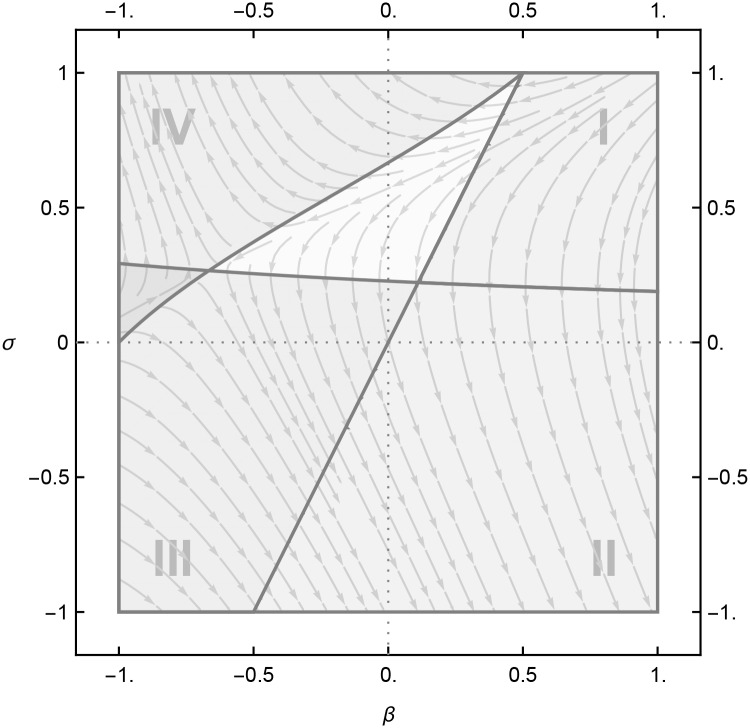
Optimal R&D, x*, conditional on *β*, *σ* and *bγ*. Arrows indicate a positive change in R&D investment for a change in either *β* or *σ*.

Fig 1 illustrates how product rivalry and technology spillovers mediate optimal process R&D investments. The two subfigures display the optimal R&D investments in *β*-*σ* space with the horizontal axis depicting knowledge spillovers (*β*), and the vertical axis, the degree of product substitution (*σ*). Subfigure (a) shows an example of low efficiency in R&D investments, assuming a high value for *bγ* (in the diagram, we assumed *bγ* = 50). The right panel in Subfigure (b) depicts the case of highly efficient R&D investments with *bγ* = 4; note that this value represents the infimum of the product of these two parameters. Below this value, the second-order condition, required to be negative for an optimal choice of R&D investment in the second stage of the game, will no longer be negative over the whole domain of *β* and *σ*. Hence, for some values of *σ* and *β* (upper left and lower right corners of the *β* − *σ* space), the second-order condition becomes positive, rendering R&D investments unbounded from above. The higher *bγ*, the closer the line, which spans the *β*-*σ*-space from left to right, to the vertical axis. This observation will also be taken into account in the empirical part of the paper.

Furthermore, if *σ* > 2*β*, the R&D levels of *x*_*i*_ and *x*_*j*_ are positively related: any change in firm *i*’s R&D investments is associated with a corresponding change in firm *j*’s R&D investments. If instead *σ* < 2*β*, any change in firm *i*’s R&D spending leads to an opposite change in firm *j*’s R&D investment.

The line connecting the points (*β* = −0.5, *σ* = 1) and (*β* = .5, *σ* = 1) in Fig 1 divides the *β*-*σ*-plane into two corresponding regions: the left region with substitutive R&D investment behavior and the right region with complementary investment behavior. Whether complementary or substitutive R&D investment behavior leads to a higher or lower optimal x*(*β*, *σ*) depends on *β* and *σ*. The line between (*β* = −1, *σ* = 0) and (*β* = 0.5, *σ* = 1) and the line close to the vertical axis—the line which is mediated by *bγ*, as explained above—separate the plane into further subregions. Due to the sensitivity of the model to *bγ*, we put our focus on the four regions, in which R&D investment reactions of firms should be clearly observable in the data. To make sure that the limits of the regions do not get blurred in our empirical study, we reduce these four regions even further. The corners marked by dashed rectangles in Subfigure (b) of Fig 1 will form the basis of our analysis.

For further clarification of the two panels in this figure: The line running from (*β* = −1, *σ* = 0) to (*β* = .5, *σ* = 1) denotes all combinations of *β* and *σ* where ∂x*/∂*σ* = 0. The second line close to the horizontal axis, separating subregions I and IV from subregions II and III, subsumes all *loci* with ∂x*/∂*β* = 0. The underlying stream plot in the two subfigures depicts the direction of the highest slope in optimal x*(*β*, *σ*) as mediated by *σ* and *β*. This leaves us with the following four major regions in the *β*-*σ* space:

- Complementary R&D investment: 0 < *dx*_*i*_/*dx*_*j*_ < 1
  - Region I with ∂x*/∂*σ* < 0 ∧ ∂x*/∂*β* < 0
  - Region II with ∂x*/∂*σ* < 0 ∧ ∂x*/∂*β* > 0
- Substitutive R&D investment: −1 < *dx*_*i*_/*dx*_*j*_ < 0
  - Region III with ∂x*/∂*σ* < 0 ∧ ∂x*/∂*β* > 0
  - Region IV with ∂x*/∂*σ* > 0 ∧ ∂x*/∂*β* < 0

This model enlightens the rationale underlying process R&D decisions by firms. Such decisions not only impact firms’ own marginal costs but also affect rival companies’ decisions by affecting their supply and demand curves *via* the contextual parameters *β* and *σ*, respectively. More precisely, an increase in R&D investments by firm *i* entails several effects: (1) a shift of firm *i*’s supply curve to the right by a magnitude of *x*_*i*_, as process innovation decreases marginal costs, (2) a reallocation of market shares as in the standard Cournot model, and (3) a countervailing effect to effect (2) because technology spillovers also reduce the representative competitor *j*’s marginal costs by a magnitude of *βx*_*i*_, thus shifting its supply curve to the right.

Whether the representative competitor *j* eventually increases (resp. decreases) its R&D investments in return, however, is unclear. This depends on the firm’s location in the *β*-*σ* space. In Region I, both technology spillovers and product rivalry are high. If firm *i* increases its R&D investments, the loss incurred by firm *j* due to the shift of its residual demand curve to the left outweighs the loss incurred by technology spillovers when firm *j* increases its R&D investments in return. Therefore, it is rational for firm *j* to also increase its level of R&D investment.

Fundamentally, in Region I, diminished demand due to product rivalry dominates the enhanced supply that results from technology spillovers. This in turn renders process R&D less attractive for any cost-reducing innovation spread over a narrower scale of production. Therefore, firm *i* as well as the representative competitor *j* have a strong incentive to diminish their research investments and the positive correlation between both firms’ R&D investments is due to the fact that each firm finds it beneficial to free ride on the other firm’s R&D.

Region II implies product complementarity with positive spillovers. An increase in process R&D by firm *i* reduces firm *j*’s marginal costs, shifting the supply curve downwards, and increases demand for product *j* by shifting its demand curve upwards due to product complementarity. These mutually consistent demand and supply effects clearly act as an incentive for firm *j* to also increase its R&D effort as a result of the increased optimal quantity $q_{j}^{*}$. Conditional on a sufficiently high cost parameter *γ*, the convexity of the R&D cost function ensures the existence of an upper equilibrium. This increases the marginal return to firm *j*’s R&D, incentivizing firm *j* to increase its R&D effort.

The positive correlation observed in Regions I and II must be distinguished from one another. In Region I, both firms reduce their R&D investments to benefit from their rivals’ efforts. Therefore, the collective level of R&D investments remains at a lower threshold, as depicted by the stream plots in Fig 1. In Region II, however, both firms find it profitable to increase their R&D efforts. Hence, it is no surprise that the maximum level of R&D investment is found in Region II.

In Region III, where products are complements with large negative spillovers, an increase in process R&D by one company will, on the one hand, dissuade the other company to produce more due to increased marginal costs, and, on the other hand, it will motivate the company to produce more due to the increased demand that stems from product complementarity. Because the upward shift in the supply curve dominates that in the demand curve, the company will decrease its output level. This in turn renders process R&D less attractive and leads to a decreased level of process R&D by the rival company. In other words, there is substitution in process R&D.

Region IV also involves mutually consistent effects, but in the opposite direction. With negative spillovers and product substitution, an increase in investment in process R&D by firm *i* shifts the supply curve upwards the demand curve downwards, reducing the optimal quantity of the rival company $q_{j}^{*}$. This renders process R&D less profitable and acts as a disincentive to invest in process R&D.

Our theoretical framework is compatible with but not identical to a series of models that link innovative activities and product market competition. This resembles the work of Aghion et al. [13], who argue that the relationship between competition and R&D activities is an inverted U-shaped relationship, implying that loose or fierce competition is detrimental to innovation. Instead, we argue that it is not only the level of competition alone that matters but also the level of spillovers.

Our theory says that the sign of the reaction function *dx*_*i*_/*dx*_*j*_ depends on the location of firm *i* and its representative competitor *j* in the *β*-*σ* space. More precisely, we aim to estimate the sign of the reaction function *f*(*x*_*jt*_) for each of the four corners of the *β*-*σ* space. In order not to run the risk that the effects of *β* and *σ* get blurred by the uncertainty of actual limits between regions, we decided to confine our empirical study as in our theoretical model even further. The two subfigures in Fig 1 illustrate that a low value of *bγ* will reduce Region IV. Since neither *b* nor *γ* were observed in our data, the actual magnitude of *bγ* remains uncertain. Likewise, the diagonal line between (*β* = −0.5, *σ* = 1) and (*β* = .5, *σ* = 1), separating Regions I and II from Regions III an IV, subsumes loci in which the sign of the derivatives with respect to *β* and *σ* are close to zero. For these reasons, we look exclusively at the data that can be classified into the dashed rectangles in Subfig Fig 1b.

Theory also warns about the stability of the reaction functions for Regions II and IV: with *sufficiently* high research costs *γ*, the reaction functions are well-behaved and lead to a stable equilibrium. This has been put forward by Henriques [14], who analyzes these conditions for the model by d’Aspremont Jacquemin [12]. In Region II, below a threshold value for research cost *γ*, the reaction functions leads to an unstable equilibrium where full specialization by one firm occurs: only one company undertakes R&D activities, whereas the other chooses to withdraw from research activities. Moreover, for even lower levels of *γ*, the second-order conditions may not be fulfilled for Region IV. Therefore,

$$\begin{array}{c} \begin{array}{c} dx_{i}/dx_{j}>0\;\text{in}\;\text{Region}\;\text{I}\\ dx_{i}/dx_{j}\geq 0\;\text{in}\;\text{Region}\;\text{II}\\ dx_{i}/dx_{j}<0\;\text{in}\;\text{Region}\;\text{III}\\ dx_{i}/dx_{j}\leq 0\;\text{in}\;\text{Region}\;\text{IV} \end{array} \end{array}$$

## 3 Empirical protocol

The empirical exercise is to estimate the R&D reaction functions between any two firms *i* and *j*, as shown in Eq (17), that is, to estimate the elasticity of R&D investment decisions *x* made by firm *i* with respect to the R&D investment of firm *j*:

$$\begin{array}{c} x_{i}=f(x_{j})+\xi_{i} \end{array}$$ (19)

To estimate Eq (19), we need financial data on R&D decisions and other firm characteristics and data that would allow us to determine both the amount of potential spillovers *β* and the level of product substitution *σ* between any two firms *i* and *j*. Data on the world’s largest corporations allow us to address these issues.

### 3.1 Computing the empirical *β*-*σ* space

The difficulty lies in measuring product substitution *σ* and technological spillovers *β* between any two firms to reveal the concealed *β*-*σ* space. Reliance on the cosine index is pervasive in the literature since [15] to measure technological spillovers [16, 17], Nesta Saviotti JIE 05. The rationale is that firms that develop competencies in similar technologies should benefit from each other’s advances in research, more so than companies that are active in entirely different fields. More recently, [1] rely on the cosine index to measure technology spillovers *and* product market rivalry.

Because theory specifies that both *σ* and *β* belong to the interval [−1; +1], reliance on the cosine index, which lies in the [0; +1] interval, is an issue. Instead, we use Pearson’s correlation coefficient *r* to compute proximity measures in the technology and product market space which produces measures of product substitution *σ* and of technological spillovers *β* that lie between [−1; +1]. Observe that Pearson’s *r* is nothing else than the cosine index computed on mean-centered values.

Concerning technological spillovers *β*_*ij*_, we proceed as follows. We use patent data to describe the firms’ portfolio of technological competencies and use the latter to measure pairwise correlations in the technology portfolio for any dyad. Patent data come from the USPTO dataset provided by the National Bureau of Economic Research [18]. This dataset contains more than 3 million US patents issued since 1963. Using information on each company’s name and year of application, we selected the firms most active in patenting using the 2000 Edition of Who Owns Whom to control for firm consolidation. Importantly, the USPTO dataset assigns each patent to several international patent technology classes (IPC). The six-digit technology classes proved too numerous, so we adopted the three-digit level, corresponding to a technological space of 120 technologies.

Let *p*_*ikt*_ be the number of patents applied for by firm *i* in technology class *k* during year *t*. Because the knowledge underlying a patent is durable for a longer time span, we assume that all patents have a life span of five years. Therefore, for a given technology *k*, we define *T*_*ikt*_ as the sum of patents over the past five years: $T_{ikt}=\sum_{\tau =0}^{4}p_{ik,t-\tau }$. We can then describe the technological profile of companies by a vector of technological competencies **T**_**t**_, where generic component *T*_*ikt*_ is the accumulated number of patents in a given technological field in a given year. Leaving the time subscript aside and writing $\tilde{\text{T}}$ as the mean-centered vector of patents **T**, technological spillovers *β*_*ij*_ between the two vectors ${\tilde{\text{T}}}_{\text{i}}$ with ${\tilde{\text{T}}}_{\text{j}}$ reads

$$\begin{array}{c} \beta_{ij}=\frac{{{\tilde{\text{T}}}^{\prime }}_{i}{\tilde{\text{T}}}_{j}}{\sqrt{{{\tilde{\text{T}}}^{\prime }}_{i}{\tilde{\text{T}}}_{i}}\cdot \sqrt{{{\tilde{\text{T}}}^{\prime }}_{j}{\tilde{\tilde{\text{T}}}}_{j}}} \end{array}$$ (20)

where the subscripts *i* and *j* denote firms *i* and *j*, respectively. Two companies that are developing competencies in the same or similar vectors of technologies are supposedly more inclined to identify, assimilate and exploit each other’s R&D findings [19], thereby benefiting from the rival’s R&D and incorporating it into their own production function and decreasing the marginal cost of production. The case of the nullity of the Pearson’s *r* implies full independence in the firms’ technology portfolios, where neither positive technological externalities nor negative pecuniary externalities can occur. A negative correlation coefficient implies that areas of relative specialisation of company *i* represent areas of relative under-investment by company *j*. Although in this case little gains from technological externalities can be expected, pecuniary externalities are likely to dominate.

Concerning product substitution, one would ideally use demand functions on particular pairs of products or even use the technological characteristics of products to measure the distances between any pair [20]. In both cases, however, data are difficult to find, especially when they need to be combined with additional information on such areas as technology spillovers and company accounts. Instead of concentrating on all types of firms, we focus on multi-product firms and argue that product substitution, or the degree of market rivalry, can be measured using the vector of sales of companies across several market segments.

One could be tempted to adopt a similar reasoning as the one above for technological spillovers. In this case, the idea is that firms competing on similar markets compete with one another. Suppose that multi-product companies can be described by a vector of sales **Y**, where generic component *Y*_*is*_ provides the amount (level) of sales by firm *i* for a given 4-digit sector segment *s*. It is then straightforward to compute Pearson’s *r* between the two vectors ${\tilde{\text{Y}}}_{\text{i}}$ and ${\tilde{\text{Y}}}_{\text{j}}$. This calculation leads to $\sigma_{ij}^{lev}$, the degree of market rivalry measured in levels:

$$\begin{array}{c} \sigma_{ij}^{lev}=\frac{{{\tilde{\text{Y}}}^{\prime }}_{i}{\tilde{\text{Y}}}_{j}}{\sqrt{{{\tilde{\text{Y}}}^{\prime }}_{i}{\tilde{\text{Y}}}_{i}}\cdot \sqrt{{{\tilde{\text{Y}}}^{\prime }}_{j}{\tilde{\text{Y}}}_{j}}} \end{array}$$ (21)

where subscripts *i* and *j* denote firms *i* and *j*, respectively, and $\tilde{\text{Y}}$ is the mean-centered vector of sales across business segments.

However, there is an issue in Eq (21) level as to whether the correlation coefficient of sales over several market segments really captures rivalry on the product market. Instead, one would use cross-product elasticities to properly grasp whether two goods are complements or substitutes. In order to approach the idea of cross-product elasticities, we transform the vector of sales **Y** in growth rates $\dot{\text{y}}$ for all companies, and then compute the correlation coefficient over the vector of growth rates as follows

$$\begin{array}{c} \sigma_{ij}^{gr}=\frac{{{\tilde{\dot{\text{y}}}}^{\prime }}_{i}{\tilde{\dot{\text{y}}}}_{j}}{\sqrt{{\tilde{\dot{\text{y}}}}_{i}{\tilde{\dot{\text{y}}}}_{i}}\cdot \sqrt{{{\tilde{\dot{\text{y}}}}^{\prime }}_{j}{\tilde{\dot{\text{y}}}}_{j}}}\times -1 \end{array}$$ (22)

Observe that the use of of growth rates implies that we multiply the Pearson’s *r* by −1. The reason for this is that two firms enjoying a positive growth rate on the same business segment are likely to have complementary products, whereas two firms competing on the product markets should cope with a negative correlation in the growth rates. Hence a positive correlation coefficient in the sales growth rates between any two firms implies product complementarity, a dimension which in our theoretical framework is depicted when *σ* < 0.

In the empirical part, our preference goes to Eq (22), although we will also conduct robustness checks using Eq (21) as a measure of rivalry on the product market side.

### 3.2 Control variables

Past research shows that R&D investment by firms is affected by factors other than the level of R&D investments of rival firms.

First, we include a proxy for the efficiency parameter *γ* in Eq (3) and define *γ*_*i*_ as the patent productivity of R&D investments (*P*/*X*)_*i*_, where *P* is the number of patents granted to firm *i* and *X* is the firm’s R&D investment. We lag this variable two years to avoid simultaneity in the relationship. Second, [21] and [22] have stressed the interdependence of firm size and R&D investments. Because large firms have an advantage in spreading the cost of research over a larger span of output, R&D investments tend to increase monotonically with size. We therefore include firm size *K* into the empirical model using the gross value of plant and equipment.

Third, strategic investment decisions also depend on financial constraints [23, 24, 25]. When returns on investments are subject to substantial uncertainty, as is the case with research activities, firms increase cash flow availability to secure in-house investment capacities as a response to the lack of external financial resources [26]. If markets were perfect, investment decisions could be financed by either internal means or external credit availability. In the presence of imperfect markets, however, limited access to external financial resources will be compensated for by increases in cash availability provided by the firm itself. This makes it easier for the company to undertake investment decisions. We therefore include the so-called liquidity ratio (*LR*), defined as the cash flow availability normalized by current liabilities. Should financial markets be imperfect, a positive association between R&D decisions *X* and *LR* should be depicted.

Because variables on firm size and financial constraints influence future decisions, we lag all control variables by one year. Moreover, we include a full vector of year dummies to account for the year-specific shocks common to all firms in the sample. Unobserved firm heterogeneity is accounted for through the use of pre-sample mean panel data models.

### 3.3 Data sources

Compustat is the source of all firm-level accounting data. The gross value of property, plant and equipment proxies firm size (*K*); the liquidity ratio *LR* and the ratio between cash flow availability and current liabilities are used to grasp financially constrained firms. Financial data, expressed originally in national currencies, have been converted into US dollars using the exchange rates provided by the Organisation for Economic Co-operation and Development (OECD). All financial data have been deflated in 2005 US dollars using the Implicit Price Deflator provided by the US Department of Commerce, Bureau of Economic Analysis.

Compiling the data from the patent and financial sources produced an unbalanced panel dataset of 308 companies observed between 1979 and 2005, yielding 5,461 firm-year observations. These come from various industries that differ in their R&D intensity (*X*/*Y*). Of all corporations, 199 belong to high-technology sectors, including Chemicals (63 firms), Electronic Equipment (54 firms), Photographic, Medical and Optical Goods (36 firms), and Industrial Machinery and Computer Equipment (46 companies), with an aggregate R&D intensity reaching 6%. There are 62 corporations in the medium-technology sectors, namely, in Transportation Equipment (32 firms), Business Services (21 firms) and Other Sectors (9 firms), with an aggregate R&D intensity of between 3% and 5%. The low-technology sector comprises 47 firms (Furniture and Fixtures, 5 firms; Paper Products, Printing and Publishing, 12 firms; Petroleum and Refining, 10 firms; Rubber, Concrete and Miscellaneous Products, 8 firms; Metal Industries, 12 firms) (Table 1).

**Table 1:** Descriptive statistics by industry (Averages, 1979-2005).

Industry	♯ Firms	♯ Obs.	*X* ^a^	*Y* ^b^	$K_{i}^{c}$	*LR* ^d^	(*X*/*Y*)^e^	♯*P* ^g^	*γ* ^h^
Furniture & Fixtures	5	85	85.4	5,215	1,635	0.146	0.016	29.1	3.681
Paper Products, Printing & Publishing	12	212	229.7	9,507	8,924	0.210	0.024	65.1	10.200
Chemicals & Allied Products	63	1,120	676.0	9,625	7,476	0.748	0.070	101.8	16.570
Petroleum Refining	10	219	374.6	60,980	59,379	0.277	0.006	150.4	4.827
Rubber, Concrete & Misc. Products	8	130	155.9	5,840	3,748	0.243	0.027	38.4	5.313
Metal Industries	12	187	89.1	6,119	4,515	0.195	0.015	23.9	13.940
Industrial Machinery & Computer Equipment	46	850	540.1	9,384	4,930	0.545	0.058	163.3	9.704
Electronic Equipment	54	982	737.8	10,116	5,909	0.904	0.073	195.3	8.861
Transportation Equipment	32	575	1,506.0	37,635	21,608	0.223	0.040	146.4	17.470
Photographic, Medical & Optical Goods	36	614	266.2	4,432	2,552	0.523	0.060	93.0	9.226
Business Services	21	355	1,420.0	27,114	41,895	0.593	0.052	249.7	13.630
Others	9	132	1,231.0	36,280	25,868	0.256	0.034	299.3	13.680
All Sectors	308	5,461	688.2	15,598	12,277	0.567	0.044	141.5	11.820

^a^ *X*: R&D expenses, in millions of 2005 US$.

^b^ *Y*: Sales, in millions of 2005 US$.

^c^ *K*: Gross Property, Plant and Equipment, in millions of 2005 US$.

^d^ *LR*: Cash flow to current liabilities ratio.

^e^ *X*/*Y*: R&D intensity.

^g^ ♯ *P*: Number of patents.

^h^ *γ*: R&D Cost Parameter: *γ* = ♯*P*/*X*.

The Cournot-type model developed in Section (1) is based on two firms located in the *β*-*σ* space. We must therefore compute all *β*_*ij*_’s and *σ*_*ij*_’s between any pair of firms—a dyad—in the sample. Because *β*_*ij*_ = *β*_*ji*_ and *σ*_*ij*_ = *σ*_*ji*_, *N* × (*N* − 1)/2 *β* and *σ* measures are produced per year, depicting the nature of competition between any two companies *i* and *j*.

**Fig 2:**
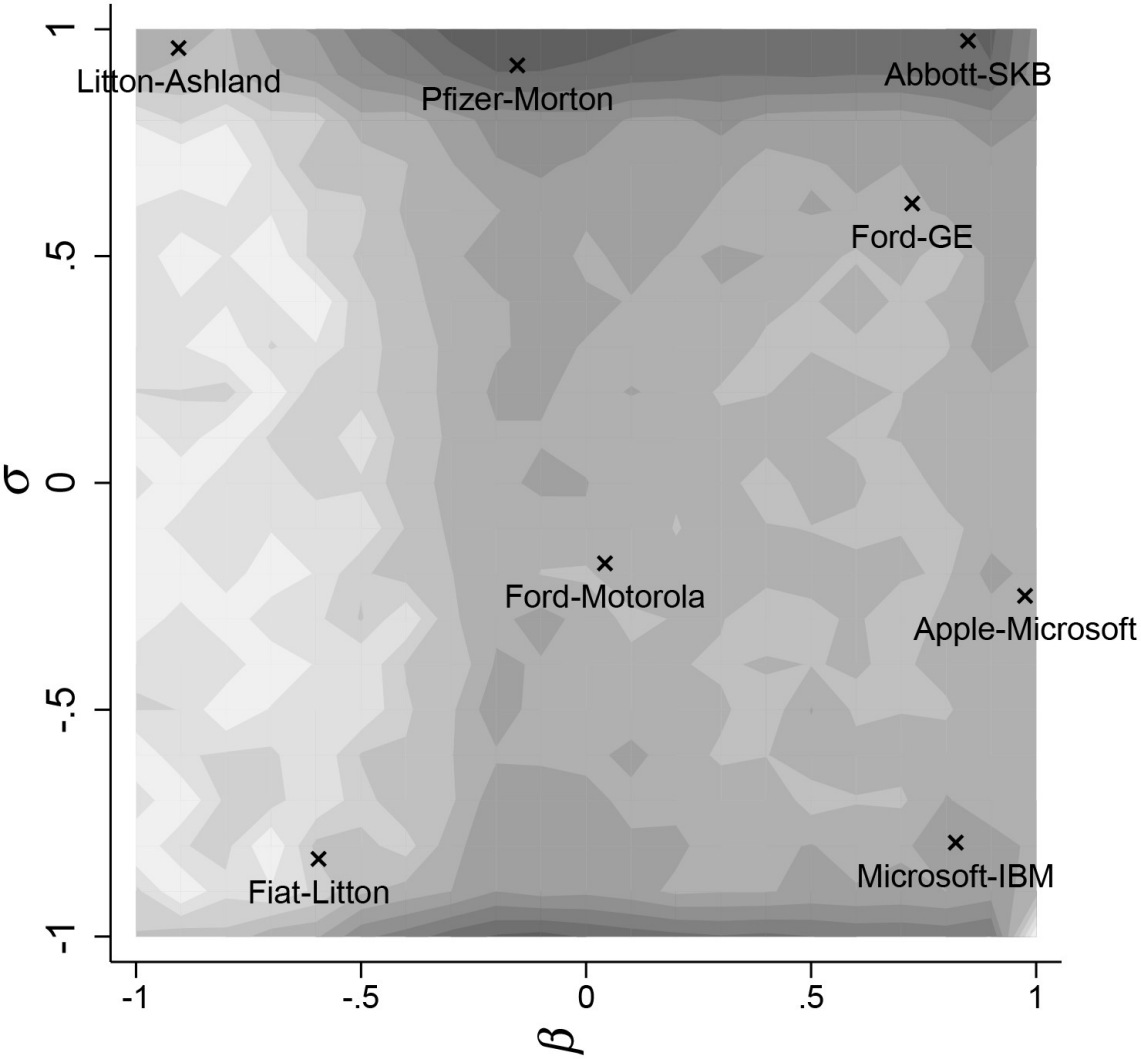
Number of dyads in the empirical *β*-*σ* space, by deciles.

Fig 2 displays the number of dyads in the obtained *β*-*σ* space, expressed in deciles. It reveals that most companies tend to avoid direct product and R&D competition because they are located in the bottom-left corner of the *β*-*σ* space. We also observe the absence of location in areas of strong technological and product rivalry, corroborating the idea that the largest corporations develop firm-specific portfolios of business lines and technological competencies.

The figure also points at specific dyads. In Region I, we find dyads in two heavily competitive markets: Abbott Laboratories and Smithkline Beecham for the pharmaceutical preparation industry and the well-known rivalry between Ford and General Electric in the automobile industry. Fierce product market rivalry is also found in the case of Pfizer (pharmaceutical preparation) and Morton International (Miscellaneous chemical products), albeit with significantly different technology portfolios. The same applies to Litton Industries Inc. (Ship & boat building) and Ashland Inc. (Chemical and allied substances). In this Region (Region IV), firms may mainly suffer from the rival’s R&D efforts in that it increases the marginal cost of production due to pecuniary externalities.

At the bottom of Fig 2, we display three dyads that have market complementarity in common. Although with a substantial level of technological overlap, market rivalry between Microsoft and Apple appears lower as a result of the presence of Apple in the hardware industry, whereas Microsoft is committed to the Prepackaged Software industry. Complementarity in products appears in the case of Microsoft (Electronic computers) and IBM (Computer Programming) with very similar technology portfolios. Because this dyad is located in Region II, the presence of positive spillovers is expected. This is the ideal location for dyads: each company benefits from the R&D executed by the other company, thus lowering its marginal cost. It also benefits from increased sales by the partner because of product complementarity. At the other extreme (Region III), we find dyads with product complementarity and dissimilar technology portfolios such as Fiat (Motor Vehicles) with Litton Industries (Ship and boat building). In this Region, the theory predicts that strategic partners suffer from each other’s R&D due to pecuniary knowledge externality.

In Table 2, we display the mean values for *β* and *σ* using the business lines provided by Compustat, which uses the standard industrial classification. We thereby distinguish dyads in which the two companies come from the same business line with respect to their main activity from those with different main business lines.

**Table 2:** Average measures of *β* and *σ*^*gr*^ for intra industry (*n*) and cross (*x*) industry dyads.

Industry	${\bar{\beta }}_{n}$	${\bar{\sigma }}_{n}^{gr}$	${\bar{\beta }}_{x}$	${\bar{\sigma }}_{x}^{gr}$	${\text{sd}}_{\beta_{n}}$	${\text{sd}}_{\sigma_{n}^{gr}}$	${\text{sd}}_{\beta_{x}}$	${\text{sd}}_{\sigma_{x}^{gr}}$
Furniture & Fixtures	0.310	0.062	0.043	-0.250	0.507	0.727	0.450	0.680
Paper Products, Printing & Publishing	0.372	-0.267	-0.025	-0.181	0.380	0.712	0.378	0.724
Chemicals & Allied Products	0.332	-0.185	0.001	-0.192	0.436	0.799	0.405	0.761
Petroleum Refining	0.581	-0.228	0.060	-0.208	0.295	0.672	0.369	0.713
Rubber, Concrete & Misc. Products	0.220	-0.065	0.011	-0.273	0.477	0.740	0.415	0.734
Metal Industries	0.101	-0.382	0.061	-0.291	0.394	0.709	0.411	0.716
Industrial Machinery & Computer Equipment	0.081	-0.304	0.028	-0.254	0.434	0.750	0.411	0.721
Electronic Equipment	0.315	-0.265	0.055	-0.232	0.432	0.769	0.412	0.728
Transportation Equipment	0.401	-0.261	0.037	-0.231	0.364	0.706	0.375	0.716
Photographic, Medical & Optical Goods	0.292	-0.343	0.111	-0.253	0.498	0.776	0.442	0.735
Business Services	0.396	-0.135	0.087	-0.229	0.460	0.828	0.432	0.771
Others	0.202	-0.340	0.131	-0.262	0.352	0.593	0.386	0.698

$\bar{\beta }$ and ${\bar{\sigma }}^{gr}$ denote arithmetic average of technological spillovers and product market rivalry, respectively.

sd: Standard deviation

The potential for technology spillovers appears to be substantially higher for companies that share the same main business line. For all intra-industry dyads ${\bar{\beta }}_{n}$ is significantly positive, with an average value of .287 whereas the cross-industry dyads have an average value of ${\bar{\beta }}_{x}=.046$. No such pattern is found for product market rivalry, where *σ*^*gr*^ is negative and averages at -.25 and -.24 for intra and cross-industry dyads. In fact, the Student t-test reveals that the difference in technological spillovers between intra and cross industry dyads is significant with a t-value of 190.00, whereas the difference in product market rivalry between intra and cross industry dyads is insignificant with a t-value of 1.42. The message of Table 2 is that: although two large, multi-product firms belonging to the same industry may be either rivals or deliver complementary products, the accumulation of technological competencies within an industry is less flexible. This is in line with Pavitt and Patel [27, p.156], who argue that contrary to the sphere of products, the sphere of technology “(…) is underpinned by quite rigid, one-to-one technology imperatives: if you want to design and make automobiles, you must know about mechanics; if you want to design and make aeroplanes, you must know about aeronautics (…)”

The above is corroborated when we look at the standard deviations of both technological spillovers and product market rivalry. In fact, the standard deviation is much lower for technological spillovers than for product rivalry. This outcome is compatible with the presence of some form of technological determinism [27], whereas the scope for market location remains wide. In other words, product variety within an industry is fully compatible with technological homogeneity.

### 3.4 Econometric specifications

The empirical model estimates the reaction function of firm *i* in its R&D investment *x*_*it*_, conditional on firm *j*’s R&D investments *x*_*jt*_. First, we enter all variables in logs, estimating the elasticity of *x*_*i*_ with respect to *x*_*j*_.

$$\begin{array}{c} x_{it}=\alpha +\omega x_{jt}+\text{B}{\text{C}}_{\text{it}}+\xi_{\text{it}} \end{array}$$ (23)

where *t* = {1979, …, *t*, …, 2005}, lower cases indicate log transformed variables, *ω* is the parameter of interest, and **B** is the vector of parameters of control variables **C**_**it**_. This econometric specification addresses three important issues, namely, firm unobserved heterogeneity, firm *i*’s decision-making process and the endogeneity of the RHS variable *x*_*jt*_.

First, unobserved variations in the characteristics of companies may influence firm R&D investments beyond and above the chief role of past R&D decisions, rival’s R&D investment, size and financial constraints. Such concealed dimensions may come from the firm’s research ties developed with private partners or/and with public research organizations, the organizational culture of the company to be located at the forefront of the technological frontier, or, among other things, the CEO’s inclination to orient a research program towards ambitious and costly objectives. Ideally, one would include a firm-fixed effect to control for such unobserved attributes. However, within-transformations are known to produce inefficient estimates when samples are small and regressors are persistent, as is the case with R&D series. As an alternative estimator we use the pre-sample mean (PSM) estimator that replaces the fixed effect by the pre-sample mean of the dependent variable. [28] show that this estimator is consistent when the number of pre-sample periods is large for the dependent variable and has better finite sample properties than the fixed effect model. Therefore, we choose to include the pre-sample mean measure of the dependent variable, and constrain the sample to include observations after 1990 only, which yields the reaction function:

$$\begin{array}{c} x_{it}=\alpha +\omega x_{jt}+\gamma {\bar{x}}_{ip}+\text{B}{\text{C}}_{\text{it}}+\xi_{\text{it}} \end{array}$$ (24)

where *t* = {1990, …, *t*, …, 2005}, and where $x_{ip}=\left(1/TP\right)\sum_{r=0}^{TP}x_{i,1989-r}$ represents the pre-sample mean, *r* = {0, …, *r*, …, *TP*} and *TP* is the number of pre-sample observations. Variable *x*_*ip*_ grasps persistent differences in R&D investments across firms and acts as a control for unobserved heterogeneity between firms.

Second, the duopoly model of the theoretical Section implies that each firm makes investment decisions based on the optimal investment of the rival company. Empirically, however, companies cope with an array of competitors so that the duopoly assumption is violated in most markets. In other words, the optimal R&D decision depends on the behavior of more than one rival only. Therefore, we assume that companies do not make inferences on their optimal R&D decisions based on each of their rivals. Similarly to [29], we assume that firms make *oblivious* R&D choices, that is, decisions on R&D investments based on the R&D decision of the *average* rival company. Specification (23) then becomes

$$\begin{array}{c} x_{it}=\alpha +\omega {\bar{x}}_{jt}+\gamma {\bar{x}}_{ip}+\text{B}{\text{C}}_{\text{it}}+\xi_{\text{it}} \end{array}$$ (25)

Third, simultaneous decisions by companies imply that if *x*_*i*_ is determined by *x*_*j*_, the opposite relationship equally holds. This mutual dependence together with the specification in Eq (24) calls for the use of additional moment restrictions that account for the correlation between endogenous variables ${\bar{x}}_{jt}$ with the error term *ξ*_*it*_:

$$\begin{array}{c} E(\xi_{it},(\begin{array}{c} {\bar{x}}_{jt-1}\\ {\bar{x}}_{st} \end{array}))=0 \end{array}$$ (26)

Note that part of the endogeneity should already be withdrawn when using ${\bar{x}}_{j}$. When the number of companies *n* is high, individual decisions of firm *i* will influence ${\bar{x}}_{j}$ only marginally, i.e. by 1/(*n* − 1). We instrument ${\bar{x}}_{jt}$ by its own lagged values and computed variable ${\bar{x}}_{st}$, which measures the average level of investments in product segment *s* at year *t*, as in the following:

$$\begin{array}{c} {\bar{x}}_{st}=\text{ln}(\frac{\sum_{j\neq i}x_{jst}}{N_{s}-1}) \end{array}$$

with *N*_*s*_ as the number of companies in business segment *s*. Hence ${\bar{x}}_{st}$ is the (log of) average R&D investment, the main business segment in which firm *i* is active, excluding firm *i*’s own investment. Therefore, variable ${\bar{x}}_{st}$ is firm-specific.

Then, model 25 can be estimated using the well-known two step procedure, where the first step regresses the endogenous variable ${\bar{x}}_{st}$ on its instruments and the second step includes predictions from the first step into the specification of interest, i.e. model (25). Four regressions are performed, one for each region in the empirical *β*-*σ* space. Based on Fig 1, we assign dyads to the four regions as follows. Region I gathers dyads in which both technology spillovers and product substitution are positive, and the marginal effect of technology spillovers dominates the marginal effect of product substitution, i.e. 0 ≤ *β*_*ij*_ ≤ 1 ∧ 0 ≤ *σ*_*ij*_ ≤ 1 ∧ *σ*_*ij*_ < 2*β*_*ij*_. Region II concerns dyads in which product substitution is negative and the effect of technology spillovers dominates the effect of product substitution: −1 ≤ *σ*_*ij*_ ≤ 0 ∧ *σ* < 2*β*. Region III concerns dyads in which both technology spillovers and product substitution are negative −1 ≤ *β*_*ij*_ ≤ 0 ∧ −1 ≤ *σ*_*ij*_ ≤ 0 ∧ *σ* > 2*β*. Region IV concerns dyads in which product substitution is positive and its marginal effect dominates the marginal effect of technology spillovers with 0 ≤ *σ*_*ij*_ ≤ 1 ∧ (*β* + 3)*σ* > *β*(*σ*^2^ + 2) + 2.

Table 3 provides descriptive statistics for each region of the empirical *β*-*σ* space. It is noteworthy that product rivalry (*σ*^*gr*^) divides dyads in two groups of similar size, where half compete on the product market (*σ*^*gr*^ > 0) and half sell complementary products (*σ*^*gr*^ < 0). By exclusively focusing on competition on the product market (*σ* ≥ 0), one actually screens out a substantial share of the strategic positioning of firms in the *β*-*σ* space.

**Table 3:** Descriptive statistics by region.

Variable	Region	♯ Dyads	Mean	Median	St.dev.	Min.	Max.
ln *x*_*i*_	1	1,060	6.097	6.075	1.520	1.692	9.238
${\bar{x}}_{j}$	1	1,060	6.288	6.307	1.307	1.743	9.238
ln *k*_*i*_	1	1,061	7.931	7.996	1.652	2.643	11.77
ln *LR*_*i*_	1	999	-1.330	-1.289	1.305	-5.881	1.906
*γ*_*i*_	1	929	-1.635	-1.513	1.182	-6.532	3.001
ln *x*_*i*_	2	1,575	5.933	5.888	1.555	-0.916	9.115
${\bar{x}}_{j}$	2	1,575	6.160	6.189	1.098	0.017	9.030
ln *k*_*i*_	2	1,575	7.839	7.939	1.623	3.174	11.77
ln *LR*_*i*_	2	1,489	-1.391	-1.361	1.313	-6.029	2.163
*γ*_*i*_	2	1,376	-1.569	-1.445	1.189	-6.532	6.116
ln *x*_*i*_	3	447	5.700	5.492	1.606	-0.192	9.007
${\bar{x}}_{j}$	3	447	5.552	5.365	1.422	1.692	8.808
ln *k*_*i*_	3	447	7.520	7.502	1.625	3.174	11.60
ln *LR*_*i*_	3	424	-1.373	-1.327	1.333	-5.881	2.163
*γ*_*i*_	3	335	-1.677	-1.532	1.341	-6.532	5.447
ln *x*_*i*_	4	1,073	5.897	5.878	1.577	-0.916	9.030
${\bar{x}}_{j}$	4	1,074	5.967	5.968	1.206	-0.916	9.030
ln *k*_*i*_	4	1,074	7.700	7.820	1.561	2.643	11.77
ln *LR*_*i*_	4	1,013	-1.406	-1.398	1.353	-6.029	1.906
*γ*_*i*_	4	947	-1.590	-1.451	1.224	-6.532	6.116

See previous Table for the definition of variables.

Region I: $2\beta_{ij}>\sigma_{ij}^{gr}\wedge 0<\sigma_{ij}^{gr}<1$

Region II: $2\beta_{ij}>\sigma_{ij}^{gr}\wedge -1<\sigma_{ij}^{gr}<0$

Region III: $2\beta_{ij}<\sigma_{ij}^{gr}\wedge -1<\sigma_{ij}^{gr}<0$

Region IV: $\left(\beta_{ij}+3\right)\sigma_{ij}^{gr}>\beta_{ij}({\sigma_{ij}^{gr}}^{2}+2)+2\wedge 0<\sigma_{ij}^{gr}<1$

Our theory predicts that *ω*, the sign of the reaction function *dx*_*i*_/*dx*_*j*_, depends on the region of the dyads in the *β*-*σ* space. Taking stock of the previous discussion, we expect the following:

$$\begin{array}{c} \begin{array}{c} H_{0}\text{:}\;\omega \leq 0\;\text{;}\;H_{a}\text{:}\;\omega >0\;\text{in}\;\text{Region}\;\text{I}\\ H_{0}\text{:}\;\omega <0\;\text{;}\;H_{a}\text{:}\;\omega \geq 0\;\text{in}\;\text{Region}\;\text{II}\\ H_{0}\text{:}\;\omega \geq 0\;\text{;}\;H_{a}\text{:}\;\omega <0\;\text{in}\;\text{Region}\;\text{III}\\ H_{0}\text{:}\;\omega >0\;\text{;}\;H_{a}\text{:}\;\omega \leq 0\;\text{in}\;\text{Region}\;\text{IV} \end{array} \end{array}$$

## 4 Results

### 4.1 Main results

Table 4 presents the results, where all sets of exclusion restrictions pass the Hansen test of validity of instruments. The results corroborate the theoretical predictions. In Regions I and II, the coefficient is both positive and significant, implying that a 1% increase in R&D investments by the representative rival company spurs the firm’s own research activities by .123% (Region I) and .112% (Region II), respectively. In Region III, a 1% increase in the representative rival firm R&D investments yields a.078% decrease in firm *i* R&D investments. In Region IV, the estimated parameter $\hat{\omega }$ remains negative, although it is less significant and of a smaller magnitude (−.047%). As mentioned earlier, the reaction function in Region IV may not reach the demand (slope *b*) and R&D conditions (*γ*) required for stability. In other Regions of the *β*-*σ* space, all *ω* parameters are larger in magnitude and more efficient.

**Table 4:** Firm-level reaction functions with contemporaneous R&D investments of the mean rival firm. IV GMM Regressions with Pre-Sample Mean ${\bar{x}}_{ip}$.

	Region I (Model 1)	Region II (Model 2)	Region III (Model 3)	Region IV (Model 4)
${\bar{x}}_{jt}$	0.123	0.112	-0.078	-0.047
	(0.028)***	(0.030)***	(0.043)**	(0.035)*
*k*_*it*_	0.253	0.277	0.354	0.359
	(0.039)***	(0.034)***	(0.055)***	(0.043)***
ln *LR*_*it*_	0.358	0.356	0.401	0.391
	(0.028)***	(0.021)***	(0.046)***	(0.024)***
ln *γ*_*it*−1_	-0.293	-0.308	-0.405	-0.297
	(0.042)***	(0.034)***	(0.057)***	(0.037)***
${\bar{x}}_{ip}$	0.430	0.430	0.322	0.371
	(0.041)***	(0.033)***	(0.055)***	(0.038)***
Constant	1.206	0.997	1.988	1.862
	(0.335)***	(0.315)***	(0.502)***	(0.367)***
Observations	650	992	276	691
R-squared	0.651	0.649	0.660	0.681
Hansen’s J	0.671	2.459	0.795	0.0337
Hansen c.p.	0.413	0.117	0.372	0.854
KP Wald F	6,678	5,058	4,274	6,419

Robust standard errors in parentheses.

*** p<0.01,

** p<0.05,

* p<0.1.

All regressions include a full vector of unreported year fixed effects. Endogenous regressors *x*_*jt*_ are instrumented using their past level and average sectoral R&D, net of the firm’s own R&D. In models 3 and 4, *x*_*jt*_ is instrumented using average sectoral R&D to satisfy the exogeneity condition imposed by the Hansen’s J test.

See Table 3 for definitions of Regions.

The parameter estimates that stem from the control variables conform to our expectations. First, firm size has a positive effect in all Regions. The liquidity ratio is significantly positively associated with levels of R&D investments in all Regions of the *β*-*σ* space. The estimated short-run elasticities span from.36% to.40%. R&D investments embody a high level of uncertainty, which may hinder private external finance. As a response to the lack of external finance, firms may accumulate cash flow to secure the financing of future research activities. Moreover, low short-term liabilities can also be a sign of low financial constraints. In both cases, either high cash flow availability or low short-term liabilities increase the liquidity ratio thereby facilitating the financing of promising research projects.

Second, Eq (14) predicts that *γ*, the R&D cost parameter, reduces optimal R&D $x_{i}^{*}$. Our results confirm that an increase in R&D costs will decrease R&D investments. This negative relationship may come from different channels. Increased R&D costs may be considered increased sunk costs, the profitability of which is highly uncertain. Increased R&D costs may also be considered increased fixed costs, increasing the minimum scale of post-innovation operations. In both cases, this may act as a counter-incentive for firms to implement new research projects, thereby decreasing overall R&D investments.

A further noteworthy outcome is the stability of all other parameter estimates that stem from the control variables. It suggests that the empirical model is correctly specified and reinforces the finding that the sign of the reaction function depends on the location in the *β*-*σ* space between any two companies, as suggested by the theoretical model.

### 4.2 Robustness checks

We perform robustness checks by addressing a number of issues related to the econometric specification. First, in order to account for the size of both firms *i* and *j*, we assume that firms decide on their R&D intensity, defined as the ratio of R&D investments *X* over firm size *K*. Therefore, we amend specification (24) as follows:

$$\begin{array}{c} \text{ln}{\left(X/K\right)}_{it}=\alpha +\omega {\bar{\text{ln}(X/K)}}_{jt}+\gamma {\bar{\text{ln}(X/K)}}_{ip}+\text{B}{\text{C}}_{it}+\xi_{it} \end{array}$$ (27)

This amendment must be understood as a way of normalizing R&D investments. By controlling for the size of both firms, it is more in line with the Cournot model of Section (1), where symmetry in cost and production is assumed. Table 5 displays the results. These remain unchanged in direction and magnitude with one notable exception. In Region IV with substantial product substitution and negative technology spillovers, the parameter estimate *ω* doubles in size, implying that a 1% increase in R&D investments by the representative rival company decreases the firm’s own research intensity by.10%.

**Table 5:** Firm-level reaction functions with contemporaneous R&D intensity of the mean rival firm. IV GMM Regressions with Pre-Sample Mean ${\bar{\text{ln}(X/K)}}_{ip}$.

	Region I (Model 5)	Region II (Model 6)	Region III (Model 7)	Region IV (Model 8)
${\bar{\text{ln}(X/K)}}_{jt}$	0.119	0.144	-0.080	-0.100
	(0.024)***	(0.024)***	(0.028)***	(0.063)*
*k*_*it*_	-0.132	-0.127	-0.157	-0.126
	(0.016)***	(0.013)***	(0.024)***	(0.016)***
ln *LR*_*it*_	0.031	0.046	0.079	0.073
	(0.021)	(0.015)***	(0.032)**	(0.019)***
ln *γ*_*it*−1_	-0.119	-0.121	-0.050	-0.086
	(0.027)***	(0.021)***	(0.040)	(0.026)***
${\bar{\text{ln}(X/K)}}_{ip}$	0.818	0.826	0.902	0.876
	(0.031)***	(0.024)***	(0.036)***	(0.028)***
Constant	0.787	0.909	1.128	0.737
	(0.183)***	(0.148)***	(0.243)***	(0.189)***
Observations	649	988	276	690
R-squared	0.844	0.842	0.860	0.818
Hansen J	0.108	0.151	3.560	1.147
Hansen cp	0.742	0.698	0.0592	0.284
KP Wald F	2,528	4,616	3,792	50.59

Robust standard errors in parentheses.

*** p<0.01,

** p<0.05,

* p<0.1.

All regressions include a full vector of unreported year fixed effects. Endogenous regressors ln(*X*/*K*)_*jt*_ are instrumented using their past level and average sectoral R&D, net of the firm’s own R&D. In models 7 and 8, ln(*X*/*K*)_*jt*_ is instrumented using average sectoral R&D to satisfy the exogeneity condition imposed by the Hansen’s J test.

See Table 3 for definitions of Regions.

The previous results are based on the use of *σ*^*gr*^ in order to detect product market rivalry between dyads. Although we strongly believe that this measure grasps important aspects of product complementarity and substitution between two firms’ array of products, an alternative way of measuring product market rivalry is simply to assess the similarity of their market positioning using *σ*^*lev*^. Table 6 displays the results for both the level of R&D investment (specification 25, left panel) and R&D intensity (specification 27, right panel). Note that the Pearson’s *r* correlation coefficient between *σ*^*gr*^ and *σ*^*lev*^ (not reported in the Table) amounts to −.023, only. This implies that the firms detected as product complements or substitutes differ from one sample to the other.

**Table 6:** Firm-level reaction functions with contemporaneous R&D decisions of the mean rival firm. Using *σ*^*lev*^ to measure product rivalry. IV GMM Regressions with Pre-Sample Mean ${\bar{x}}_{ip}$ and ${\bar{\text{ln}(X/K)}}_{ip}$, respectively.

	R&D Investments				R&D Intensity			
	Region I (Model 9)	Region II (Model 10)	Region III (Model 11)	Region IV (Model 12)	Region I (Model 13)	Region II (Model 14)	Region III (Model 15)	Region IV (Model 16)
${\bar{x}}_{jt}$	0.393	0.212	-0.129	-0.030				
	(0.050)***	(0.046)***	(0.075)**	(0.021)*				
*k*_*it*_	0.243	0.231	0.301	0.232	-0.101	-0.095	-0.114	-0.102
	(0.033)***	(0.026)***	(0.062)***	(0.025)***	(0.010)***	(0.009)***	(0.015)***	(0.010)***
ln *LR*_*it*_	0.274	0.300	0.229	0.300	0.047	0.051	0.032	0.038
	(0.022)***	(0.016)***	(0.046)***	(0.016)***	(0.011)***	(0.011)***	(0.015)**	(0.011)***
ln *γ*_*it*−1_	-0.250	-0.246	-0.184	-0.244	-0.116	-0.120	-0.090	-0.125
	(0.034)***	(0.027)***	(0.049)***	(0.024)***	(0.016)***	(0.016)***	(0.021)***	(0.016)***
${\bar{x}}_{ip}$	0.502	0.543	0.532	0.550				
	(0.035)***	(0.027)***	(0.065)***	(0.025)***				
${\bar{\text{ln}(X/K)}}_{jt}$					0.193	0.138	-0.038	-0.058
					(0.021)***	(0.022)***	(0.018)***	(0.042)*
${\bar{\text{ln}(X/K)}}_{ip}$					0.782	0.837	0.933	0.908
					(0.023)***	(0.019)***	(0.021)***	(0.021)***
Constant	-0.935	-0.012	1.445	1.800	0.542	0.486	0.626	0.483
	(0.459)**	(0.433)	(0.566)**	(0.264)***	(0.183)***	(0.187)***	(0.200)***	(0.199)**
Observations	888	1,471	265	1,719	1,506	1,804	585	1,708
R-squared	0.699	0.700	0.732	0.706	0.840	0.828	0.859	0.812
Hansen’s J	1.363	0.414	0.373	0.207	0.00214	1.483	0.524	1.566
Hansen c.p.	0.243	0.520	0.541	0.649	0.963	0.223	0.469	0.211
KP Wald F	188.6	302.4	45.14	20,017	7,614	8,805	3,966	119.7

Robust standard errors in parentheses.

*** p<0.01,

** p<0.05,

* p<0.1.

All regressions include a full vector of unreported year fixed effects. Endogenous regressors *x*_*jt*_ are instrumented using their past level and average sectoral R&D, net of the firm’s own R&D. In models 11 and 12, *x*_*jt*_ is instrumented using average sectoral R&D to satisfy the exogeneity condition imposed by the Hansen’s J test. Endogenous regressors ln(*X*/*K*)_*jt*_ are instrumented using their past level and average sectoral R&D, net of the firm’s own R&D. In models 15 and 16, ln(*X*/*K*)_*jt*_ is instrumented using average sectoral R&D to satisfy the exogeneity condition imposed by the Hansen’s J test.

See Table 3 for definitions of Regions.

Although being larger in magnitude, the estimated elasticities comply to our hypotheses, corroborating the idea that the reaction function in strategic investments may be either positive or negative, depending on the type of competition between any two firms. In Regions I and II (left panel), the set of elasticities exceeds.3 and.2%, respectively, whereas in Region III the negative elasticities amounts to.13%. Only in Region IV we observe a decline in the elasticity. Again, the stability of all other parameter estimates that stem from the control variables reinforces the robustness of the theoretical prediction. Also the right panel of Table 6 conforms to the theoretical predictions. Hence, our results are robust to both ways of measuring product market rivalry.

In Table 7, we classify dyads in an alternative way. First, we keep observations only for dyads for which we have at least 8 years of observations, that is, when both *σ* and *β* are measured. Second, we compute the median region of the dyads, ruling out cases where two median regions are being computed. Third, we assign the dyads to the median region for the entire time span. This procedure is tantamount to assuming that competition in the *β*-*σ* space between any two firms changes only slowly overtime. The left panel displays the results using *σ*^*gr*^, the right panel shows the results obtained when using *σ*^*lev*^.

**Table 7:** Firm-level reaction functions with contemporaneous R&D decisions of the mean rival firm. Characterizing dyads by their median Region, for both *σ*^*gr*^ and *σ*^*lev*^ to measure product rivalry. IV GMM regressions with Pre-Sample Mean ${\bar{x}}_{ip}$ and ${\bar{\text{ln}(X/K)}}_{ip}$, respectively.

	$\sigma_{ij}^{gr}$				$\sigma_{ij}^{lev}$			
	Region I (Model 17)	Region II (Model 18)	Region III (Model 19)	Region IV (Model 20)	Region I (Model 21)	Region II (Model 22)	Region III (Model 23)	Region IV (Model 24)
${\bar{x}}_{jt}$	0.075	0.083	-0.094	-0.206	0.219	0.141	-0.094	-0.089
	(0.022)***	(0.024)***	(0.026)***	(0.047)***	(0.023)***	(0.055)**	(0.028)***	(0.023)***
*k*_*it*_	0.509	0.240	0.688	0.513	0.117	0.316	0.450	0.346
	(0.039)***	(0.029)***	(0.048)***	(0.052)***	(0.036)***	(0.034)***	(0.042)***	(0.038)***
ln *LR*_*it*_	0.366	0.368	0.395	0.328	0.284	0.380	0.353	0.338
	(0.024)***	(0.020)***	(0.025)***	(0.029)***	(0.028)***	(0.019)***	(0.035)***	(0.022)***
ln *γ*_*it*−1_	-0.194	-0.316	-0.287	-0.300	-0.234	-0.283	-0.363	-0.355
	(0.034)***	(0.033)***	(0.046)***	(0.037)***	(0.030)***	(0.031)***	(0.046)***	(0.039)***
${\bar{x}}_{ip}$	0.288	0.551	0.203	0.207	0.615	0.444	0.372	0.453
	(0.035)***	(0.029)***	(0.040)***	(0.051)***	(0.034)***	(0.032)***	(0.036)***	(0.034)***
Constant	0.376	0.788	0.283	2.085	0.770	0.355	1.148	1.523
	(0.284)	(0.272)***	(0.417)	(0.535)***	(0.298)***	(0.526)	(0.394)***	(0.328)***
Observations	531	1,196	447	418	744	1,216	358	915
R-squared	0.782	0.713	0.746	0.767	0.700	0.655	0.734	0.708
Hansen’s J	1.339	2.535	1.829	0.597	1.440	1.900	0.399	3.397
Hansen c.p.	0.247	0.111	0.176	0.440	0.230	0.168	0.527	0.0653
KP Wald F	8,624	5,002	673.1	4,490	8,199	103.7	7,794	10,713

Robust standard errors in parentheses.

*** p<0.01,

** p<0.05,

* p<0.1.

All regressions include a full vector of unreported year fixed effects. Endogenous regressors *x*_*jt*_ are instrumented using their past level and average sectoral R&D, net of the firm’s own R&D. In models 11 and 12, *x*_*jt*_ is instrumented using average sectoral R&D to satisfy the exogeneity condition imposed by the Hansen’s J test.

See Table 3 for definitions of Regions.

In sum, all previous remarks hold: the switch in the sign of the elasticities *ω* is maintained, differing between Regions I & II on the one hand and Regions III & IV on the other. The magnitudes may vary but they barely exceed.2%, implying that although the reaction functions of the companies significantly affect firm strategic investment, more of the variance must be accounted for by the firms’ own characteristics. The remaining parameters seem stable and conform to our expectations.

In the next two tables, we focus on parameter *ω* by exclusively using Model (24). Recall that thus far, we have assumed that firms make *oblivious* decisions based on the *average* rival company. We now define the rival company according to different percentile values: the 1^st^ decile; the 1^st^ quartile; the median; the 3^rd^ quartile, and the last decile. Table 8 displays the results. The main finding is that the set of hypotheses is thoroughly corroborated, irrespective of where in the distribution of R&D investments the rival company lies. No specific pattern is found in the size of the elasticity (the slope of the reaction function) and the location in the distribution of R&D investments of the rival company.

**Table 8:** Estimated R&D elasticities for different definitions of the rival firm.

	Region I	Region II	Region III	Region IV
*H*_0_	$\frac{dx_{i}}{dx_{j}}\leq O$	$\frac{dx_{i}}{dx_{j}}<O$	$\frac{dx_{i}}{dx_{j}}\geq O$	$\frac{dx_{i}}{dx_{j}}>O$
*H*_*a*_	$\frac{dx_{i}}{dx_{j}}>O$	$\frac{dx_{i}}{dx_{j}}\geq O$	$\frac{dx_{i}}{dx_{j}}<O$	$\frac{dx_{i}}{dx_{j}}\leq O$
${\bar{x}}_{jt}$	0.123	0.112	-0.078	-0.047
	0.028	0.030	0.043	0.035
	0.000	0.000	0.033	0.091
$x_{jt}^{p10}$	0.124	0.110	-0.082	-0.046
	0.028	0.031	0.044	0.035
	0.000	0.000	0.032	0.092
$x_{jt}^{p25}$	0.124	0.109	-0.081	-0.046
	0.028	0.030	0.044	0.035
	0.000	0.000	0.031	0.091
$x_{jt}^{p50}$	0.121	0.105	-0.079	-0.046
	0.027	0.029	0.043	0.035
	0.000	0.000	0.032	0.091
$x_{jt}^{p75}$	0.123	0.116	-0.076	-0.048
	0.028	0.031	0.042	0.036
	0.000	0.000	0.035	0.091
$x_{jt}^{p90}$	0.126	0.132	-0.075	-0.050
	0.028	0.034	0.042	0.037
	0.000	0.000	0.035	0.092

Robust standard errors in parentheses. *** p<0.01, ** p<0.05, * p<0.1. All regressions include a full vector of unreported year fixed effects. Endogenous regressors *x*_*jt*_ are instrumented using their past level and average sectoral R&D, net of the firm’s own R&D. For Regions III and IV, *x*_*jt*_ is instrumented using average sectoral R&D to satisfy the exogeneity condition imposed by the Hansen’s J test.

See Table 3 for definitions of Regions.

Table 9 provides the estimated set of *ω* for the whole *β*-*σ* space, using Model (24).

**Table 9:** Estimated elasticities in the *β* − *σ* space.

*β*					
*σ*	]−1.0; −.6]	]−.6; .−.2]	]−.2; +.2]	]+.2; +.6]	]+.6; +1.0[
[+.6; +1.0[	-1.153	.026	-.086	.016	.085
	(.715)	(.058)	(.040)	(.044)	(.049)
	[.053]	[.329]	[.017]	[.361]	[.041]
]+.2; +.6]	-.365^a^	-.166	.036	.087	.062
	(.269)	(.104)	(.061)	(.064)	(.063)
	[.088]	[.055]	[.277]	[.085]	[.163]
[−.2; +.2]	-.250^a^	-.034	-.140	.029	.066
	(1.101)	(.082)	(.057)	(.061)	(.062)
	[.410]	[.339]	[.007]	[.319]	[.145]
]−.6; −.2]	-.400	-.086	-.056	.038	.040
	(.221)	(.097)	(.055)	(.058)	(.058)
	[.035]	[.187]	[.153]	[.257]	[.246]
]−1.0; −.6]	-.229	-.139	-.041	.080	.160
	(.136)	(.048)	(.035)	(.037)	(.033)
	[.047]	[.002]	[.123]	[.015]	[.000]

Robust standard errors in parentheses. One-tailed critical probability value in brackets. All elasticities are obtained from OLS with firm *i*’s R&D *x*_*it*_ as the dependent variable, and rival’s R&D ${\bar{x}}_{j}$, capital ln*k*_*i*_, liquidity ratio ln*LR*_*i*_, pre-sample mean *γ*_*i*_ and a full vector of year fixed effects.

^a^ To preserve a minimum degree of freedom, this model exclude capital ln*k*_*i*_ and include a linear time trend.

From a purely qualitative point of view, Table 9 corresponds to Fig 1 derived from the theoretical model. We observe that the left (respectively right) column provides consistently negative (resp. positive) estimates, yet efficiency is not always achieved. Although highly appreciative, these results also corroborate the relevance of the theoretical model.

## 5 Conclusion

We have developed an *n*–firm oligopoly model with firms deciding on optimal process R&D and output under different settings of product substitution and research spillovers. Our model highlights situations in which the R&D of firms can be positively correlated. The sign of the effect on a firm’s R&D investment reacting on the R&D investment decision of a representative competitor depends on the joint conditions of product substitution and research spillovers. We have identified four types of environments in terms of the level of product substitution and spillovers. We then test the prediction of the model on the world’s largest manufacturing corporations. Assuming that firms make *oblivious* R&D investments based on the R&D decision of the average rival company, we develop a dynamic panel data model that accounts for the endogeneity of the decision of the mean rival firm. The results corroborate the validity of the theoretical model.

An important policy implication is that policies that support private R&D investments should take into account the environment of the targeted companies with respect to product market rivalry and technological externalities. If the objective of policy makers is to encourage private R&D—as is the case in most developed countries—the degree of product rivalry and technological externalities will determine such policies’ effectiveness.

With fierce competition on the product market (high *σ*) and positive technology spillovers (high *β*), our model unambiguously supports policies that enforce knowledge appropriation (Region I). When companies supply complementary products (negative *σ*), our model suggests that increased technology spillovers (positive *β*, Region II) and reduced pecuniary externalities (negative *β*, Region III) provide an incentive for firms to invest in process R&D. As firms in Region II benefit from their own and other firms’ process R&D, an increase in *β*, i.e., higher technology spillovers, will increase process R&D investment. In Region III, an increase in *β* (fewer negative externalities) also increases process R&D investment of firms. Hence, any policy, such as a cluster policy that aims to increase spillovers, should be accompanied by ensuring the supply of highly skilled labor—particularly with regard to Region III.

Region IV is more challenging with respect to policy recommendations. Skill-biased technical change induces negative pecuniary externalities by increasing other firms’ marginal costs due to higher equilibrium wages as the demand for skilled labor increases. According to our model, any attempt to reduce pecuniary externalities (to increase *β*) eventually reduces the incentives for firms to invest in R&D. Therefore, although generally viewed as a source of inequality among workers and increased marginal costs for all companies, skill-biased technical change may also partially restore incentives in process R&D for firms that compete in the same markets.

There are various extensions to this research avenue. First, one needs to extend the model to product innovation, which negatively influences product rivalry. Our intention is to develop a three-stage Cournot model with horizontal product differentiation in which firms first decide on their location in the product market space and then invest in process R&D. Product R&D aims at lowering rivalry on the product market, thereby changing the firm’s incentives for process R&D. Second, we intend to apply the model to the case of public policies that support the demand for specific goods. The effect on the firm’s incentives to invest in product and process R&D remains unclear. However, at a time of substantial public support in favor of environmentally friendly goods, a better understanding of the underlying mechanisms at work is at need.

## Appendix A: Welfare analysis

Similar to d’Aspremont and Jacquemin [18], let us define welfare *W*(*Q*) as the sum of consumer and producer surplus assuming equilibrium values *x*_*i*_ = x*, *p*_*i*_ = *p*, and *q*_*i*_ = q* ∀*i* = 1, …, *n*. As the demand schedule represents a linear, downward sloping line, consumer surplus increases with output Q*. Because R&D investments affect output positively, by reducing marginal costs, *C*^Σ^, in Eq (6), an increase in x* increases Q*, and thus consumer surplus.

Adding profits of the *n* firms yields the producer surplus *PS*(Q*). From the first-order condition in Eq (13), it follows immediately that a higher x* has to coincide with a higher Q*, and therefore, with a higher producer surplus, ceteris paribus. The eventually arising R&D level in equilibrium depends on the returns to R&D investments. The marginal returns to R&D investments diminish and the efficiency of R&D, represented by the associated parameter *γ*, discourages R&D investment even more.

On these grounds, we are now able to evaluate welfare effects within the *β*-*σ*-space. From above, we conclude that an increase in x* increases q*, which is tantamount to saying that overall welfare increases with total R&D investments, unless the marginal cost of an additional unit of R&D exceeds its marginal returns. Hence, Fig 1, which depicts equilibrium R&D investment x* given *β* and *σ*, mimics the movement of welfare in this domain.

Looking at the four regions using the limits with respect to *β* and *σ*, i. e. lim_*β*−>*l*_ ∧ lim_*σ*−>1_ for all permutations of *l* ∈ {−1, 1}, the resulting welfare levels for *n* = 2 converge to:

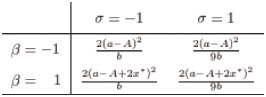

Consequently, the welfare will be highest for positive technology spillovers (*β* = 1) having complementary products (*σ* = −1) and the lowest for negative technology spillovers (*β* = −1) with homogeneous products (*σ* = 1).
