# Supplementary material for: Competition and private R&D investment
Source: PLoS One. 2020 May 27;15(5):e0232119. doi: 10.1371/journal.pone.0232119 (PMC7252648; doi:10.1371/journal.pone.0232119)
Supplement: Attachment — Submitted filename: PLOS_One_Author_Response.docx [file pone.0232119.s001.docx]

Author response to Editor and Referees

# General Letter for resubmission

Dear Editor,

My co-author and I shall like to thank you for giving us the opportunity to revise our work in order to resubmit it to be considered for publication in PLOS ONE. We shall also like to thank the two anonymous referees for their time and constructive comments. We have enjoyed their careful reading and we have tried to address their suggestions and incorporate them in the new version that we hereby submit to your scrutiny.

The new version is substantially different from the former. The main changes are the followings:

1. The introduction takes account of the remark by the Editor that we should be more explicit about how our paper relates to the former literature on spillovers and R&D investment. See our comment below (last point raised by the editor, and how we substantially changed our introduction. We now explicitly explain how our paper is positioned in the literature, namely relative to Bloom, et al. (2013).

2. The theoretical part is most similar but has incorporated all the suggestions made by the referees.
3. The empirical part has been entirely rewritten. The reason for this is the referees’ comments on the way we define corners in the paper, and repeated remarks by peer fellows who commented on the fact that our measure of product rivalry could be improved. We propose a new one in the paper, leading us to entirely rerun the empirical analysis which is now entirely new. We insist on the fact that all the results are qualitatively strictly identical to our previous conclusions.

As for data and programme availability, this paper uses both public (USPTO patent database provided by the NBER, available at no costs) and private data delivered by Compustat. We are ready to disclose all computation of indices using the patent data. However, we are not authorized to disclose firm-level information using Compustat. We would be very happy to fully cooperate with the Editor and use their advice to make the final dataset publicly available.

It goes without saying that all intermediate and final regression files using Stata programs will be made publicly available.

We hope that this new version will meet the requirement of PLOS ONE for publication.

With best wishes, we are

Prof. Thomas Grebel,

Prof. Lionel Nesta

# Specific Response to comments

# Editor

Thank you for submitting your manuscript to PLOS ONE. After careful consideration, we feel that it has merit but does not fully meet PLOS ONE’s publication criteria as it currently stands. Therefore, we invite you to submit a revised version of the manuscript that addresses the points raised during the review process.

Referees consider that the paper is well written and interesting, however, they raise some concerns about the structure of the game (justifications) and inconsistencies in notation. They consider the research question interesting, however, they find that the paper is not publishable in its present form. They recommend:

(i) Justify the restriction of the R&D reaction function in the empirical specification;

Author Response: The restriction of the R&D reaction function in the empirical specification is now motivated in the theoretical part as well as in the empirical section. We insist on the linear form of optimal R&D of the focus firm (equation 17) and echo it in the empirical form (equation 19), including an error term to insist on the fact that it is an empirical model.

(ii) discuss and justify the structure of the game;

Author Response: The structure of the game is justified all throughout the referenced literature, ranging from the seminal paper by d’Aspremont and Jacquemin (1988) to Bloom et al (2013). It also echoes the model by Strandholm, et al on how to foster environmentally friendly R&D investment. All these references, toghether with others, are mentioned in the text and display the structure of the game is the same fashion as we.

(iii) check reaction function in equation 16;

Author Response: The reaction function in equation 16 has been checked and rearranged according to reviewer 2’s request.

(iv) check inconsistencies in notation;

Author Response: We have checked the inconsistencies and corrected them.

(v) conduct a robustness check;

Author Response: We have conducted additional robustness checks. Initially, the paper was already covering quite an extensive number of robustness checks. However, we have performed additional ones. Especially, we have moved on to include other measures of product complementarity. Indeed, there was an issue as to whether the correlation coefficient of sales over several market segments really captures rivalry on the product market. Instead, one would use cross-product elasticities to properly grasp whether two goods are complements or substitutes. In order to approach the idea of cross-product elasticities, we transform the vector of sales in growth rates for all companies, and then compute the correlation coefficient over the vector of growth rates. The idea is that two firms enjoying a positive growth rate on the same business segment are likely to have complementary products, whereas two firms competing on the product markets should cope with a negative correlation in the growth rates. Hence a positive correlation coefficient in the sales growth rates between any two firms implies product complementarity.

We have decided to opt in favour of this measure of product complementarity with two major consequences. First, because growth rates are more demanding in data availability, persistency in time series, etc., the number of observation has drop. Second, all results are new, and the second part of the paper is entirely new. Figure 2 then appears new, with new dyads being depicted. Yet, table 7 shows that, whether we measure product market rivalry based on growth rates or in levels does not alter results.

A major conclusion from this work is also that our results appear very robust across all the various experimentations we have performed. We refer also to the former results when writing this.

(vi) consider developing a welfare analysis.

Author Response: We developed a brief welfare analysis and put it in the appendix, as we think that this does not relate to the core of the paper. We additionally cite further literature in which a detailed welfare analysis related to this topic can be found. We do not think that the welfare analysis is part of the issue we raise. We see no objection in cancelling this part from the paper. However, we leave it to the Editor, and the referees, to decide on this particular issue.

In addition, I believe the paper should further discuss the literature related to R&D with spillovers. For instance, Strandholm et al. (2018- Resource and Energy Economics, Volume 53, August 2018, Pages 133-146) examine a similar model but consider the effect of regulation on investment in R&D with spillovers.

Author Response: We have included additional references in the paper. We mainly think of Strandholm. Moreover, we agree with the Editor that we should better embed our paper within this literature. Therefore, we insist from the introduction onward on the contribution by Bloom, et al. as follows:

*A striking outcome of the recent paper by Bloom, et al; (2013) is that the relationship between a firm's own R&D and that of a product market rival is ambiguous. The slope of the R&D reaction function, whether positive or negative, depends on how the research effort by the rival company affects the profitability of the firm's own R&D. Our intuition is that when studying R&D reaction functions, one must first determine the context within which any two firms compete in terms of technology spillovers and product market rivalry. This mix determines whether the R&D investments by two companies are strategic complements or substitutes.*

We have decided not to include the work by Robert Wieser (2005) “R&D, Productivity and Spillovers: empirical evidence at the firm level”, Journal of Economic Surveys 19(4):587-621. This paper envisage the effect of spillovers on productivity in order to analyse the difference between the private and social rate of return of private research. This is clearly not the purpose of our paper.

## Reviewer 1.

Reviewer #1: Summary:

This paper considers the countervailing effects of technology spillover on process innovation and product competition in identifying the optimal investments in research and technology (R&D) by differentiated firms. Extending Bondt and Veugelers (1991), the authors model a two stage model, in which at the first stage, firms independently and simultaneously choose their investment levels in cost-reducing technology (which may increase or decrease the production cost of rival firms depending on the direction of spillover), and at the second stage, after observing the R&D investments of all firms, firms engage in Cournot competition by independently and simultaneously produce output (which can be substitutes or complements to each other). Using data from USPTO and Compustat, the authors estimate process innovation and product differentiation of 315 companies, and empirical results validates the internal consistency of the theoretical model.

Overall comments:

The paper is well written, with closely connected theoretical and empirical sections, and results are succinctly explained. On one hand, I think the paper will be more complete if a welfare analysis is also incorporated in sections 1 and 2. On the other hand, I would suggest stipulating the order of the game before formally discussing the model, which may probably be visualized with an extensive form of the game tree.

Author Response: See previous response to point (i).

Specific comments:

[Line 68] Please double check the summation for why the technology spillover term beta would enter into the sum quantity Q. Also, I am curious at the i =/= 1 beneath the summation operator. Do you mean j =/= i?

Author Response: Thanks for this hint. It was a typo and we corrected it.

[p.29 – 30] Footnotes 9, 10, 11, and 20 should capitalize r&d as R&D, similar to footnotes 14, 15, and 18.

Author Response: We have corrected it.

[Line 89] May I assume gamma > 0 as a regularity condition?

Author Response: We clarified that gamma >0.

[Equation 4] You miss out x_j before the closing parenthesis in the marginal cost.

Author Response: We corrected this typo in line with the comment of reviewer #2 who remarked that we should not change notations. Therefore, we now use the reduced from in equation (4), i.e C_i instead of A-x­_i-beta Sum of x­_j.

[Equation 10] May include an additional step before to show how you arrive at this result by Chain rule.

Author Response: We now address the relevant equations so that it should be clear how to derive this equation.

[Line 93] Should it be the firm’s profit maximization problem?

Author Response: Yes, it should. We have corrected this typo.

[Line 110] You can remove the preposition “with” and just begin the sentence “Inserting…”.

Author Response: We removed this preposition.

[Line 124] How do you identify the representative competitor in the theoretical model with n firms? Do you assume symmetric reactions (with uniform parameters A, b, beta, gamma, and sigma) so that firm i responds to the R&D investments of all other N – 1 firms as if one firm represents the unanimous behaviour of all other firms (and similarly for second stage output competition)?

Author Response: We added a small paragraph pointing out the symmetry of reactions.

*As we focus on strategic R&D investment behavior, we now investigate the reaction function* $R_{i}{(x}_{j})$ *with varying values of σ and β while assuming symmetric reactions with uniform parameters a, A, b, γ, β and σ. This implies that firm* $i$ *responds to the R&D investments of the remaining* $(n - 1)$ *firms.*

[Lines 133 - 4] Have you confused the horizontal and vertical axes?

Author Response: We clarified the possible misunderstanding.

[Line 178] Add a space between “investments” and “and”.

Author Response: This has been corrected.

[Line 182] Should say “both firm i and firm j”?

Author Response: This has been corrected.

[Line 218 – 26] I think sign homogeneity is still preserved if you take semi-closed bounds (-0.5, +1.0].

Author Response: See response to reviewer 2 on her/his first point.

[Line 186] Can you identify this cutoff gamma in a footnote, and a numerical estimation based on b times gamma = 100 (perhaps a lower cutoff and also an upper cutoff based on what you mention in lines 232 – 6)?

Author Response: Now, we have two subfigures with different b\gamma values (Figure 1) and we point out that b\gamma must be equal or greater than 4 in order to ensure the second order condition of the maximization problem to hold. With b\gamma -> Infinity, the line will coincide with the horizontal axis. We added this information in the text where we explain the two subfigures.

[Line 285] Do you mean competing in similar markets or competing on similar products?

Author Response: We mean similar products. This has been corrected.

[Line 311] Your present theoretical model only discusses process innovation, relegating product innovation to an extension of this paper (line 684). Could you please clarify here?

Author Response: This has been clarified. As you say, the model only discusses process innovation. We deleted “product innovation” from the text.

[Lines 368, 373, and 375] Sometimes you use the dash and sometimes you use the stroke for beta – sigma. Please make its use consistent throughout the paper.

Author Response: This has been done.

[Lines 389 and 400] What is the difference between “pecuniary externalities” and “pecuniary knowledge externality”?

Author Response: There is none in the context of this paper. We have deleted “knowledge” in line 400.

[Line 461] Can you explain your choice of different lags?

Author Response: The Hansen test in the respective tables indicates that the instruments we use are valid instruments. This test also checks, after differencing variables during the GMM procedure, whether an AR2 process is prevalent. Since the p-value suggests that the null hypothesis of “no AR2 process” cannot be rejected, instruments and therefore the choice of lags are valid. Including more lags did not change results of our GMM regressions.

[Lines 463 – 470, and 475] Please double check the signs of all regions with reference to lines 218 – 226.

Author Response: We apologize. We had updated the figure but not the text. Now, it is consistent.

[Line 484] Should add apostrophe that reads “firm i’s R&D investments”.

Author Response: Has been added.

[Line 684] Are you talking about vertical and/or horizontal differentiation for product R&D?

Author Response: We have a model of horizontal product differentiation in mind. We added this information in the text.

# Reviewer 2.

Reviewer #2: This paper develops and tests a model describing the relationship between the degree of product rivalry and R&D spillovers on the level of R&D chosen by a firm. The model develops an R&D reaction function where R&D can be either strategic substitutes or strategic complements based on the level of product rivalry and the R&D spillover. The empirical specification corroborates the theoretical results for each of the regions specified.

Comments:

1. The Regions that define the slope of the R&D reaction function come from the derivatives of firm i’s R&D investment and sigma or beta. When these regions are defined for the empirical specification (paragraph starting page 215), the regions are then restricted to 4 rectangles without justification. A figure showing how these regions change given different b*gamma values may help justify the cutoffs for these regions (showing that these "corners" are sufficiently in each of the regions).

Author Response: In this version, we have decided to redefine all region corresponding strictly to the theoretical model. The [.6;1] intervals have become more complex to define but we do provide information on their definition below Table 3 and in the core of the text, lines 528-538.

2. In the abstract, the game is described as two-stages, however this structure is not described in the model section. A formal description of the timing of the model would help the reader. Other specific comments about the model are as follows:

Author Response: We added a small paragraph before the “Output-Stage”

*Altogether, the structure of the game is as follows. In the first stage, firms choose optimal R&D investments. In the second stage, firms decide on optimal production quantity. The firm's maximization problem is solved by backward induction. Therefore, we first consider the output stage and thereafter the R&D stage.*

2a. The reaction function given in equation (16) should be derived directly from the first-order condition from the first stage, and should not be a function of firm j’s optimal investment. Given the importance of this equation, its derivation warrants a more step-by-step approach.

Author Response: We derived the reaction function from the respective profit function, and added clarifying equations.

2b. To aid in the reading of the reaction function, it may be useful to describe equation (17) as a standard linear equation of the form R_i(x_j)=intercept + slope * x_j.

Author Response: We have changed the reaction function to the usual form with intercept and slope in equation 17 and comment it explicitly below.

2c. There are some inconsistencies in notation, particularly Q is defined different on lines 68 and 95, the -i moves from a superscript on C in equation (7) to a subscript in line 100, and an i subscript seems to be missing from the p in equation (2).

Author Response: We corrected all these typos.

2d. Moving from the objective function in equation (4) to the first-order condition in equation (5) changes notation. In (4), p_i is used in its reduced form, while in (5) the derivative of the function is used, while C_i is treated the opposite way.

Author Response: Now, we use the reduced form in equation (4). In equation (5) we have to insert the full form of p_i in order to derive the first-order condition in output stage.

3. As a robustness check, it may be useful to changing the representative firm’s R&D x_j from an average of the competitor’s R&D to that of the R&D leader in the industry (measured either by expenditure or intensity).

Author Response: We beliewe that table 8 already does this job, by choosing the R&D value of the rival firm at the 10^th^ though the the 90^th^ decile. The stability of the parameter estimate is a sign of robustness. We have also experimented for additional percentile of the rival company, namely the 1^st^, the 5^th^, the 95^th^ and the 99^th^ percentiles. The results are displayed below.

| pct | Region I | Region II | Region III | Region IV |
| --- | --- | --- | --- | --- |
| mean | 0.123 | 0.112 | -0.078 | -0.047 |
|  | 0.028 | 0.030 | 0.043 | 0.035 |
|  | 0.000 | 0.000 | 0.033 | 0.091 |
|  |  |  |  |  |
| p01 | 0.124 | 0.112 | -0.082 | -0.046 |
|  | 0.028 | 0.032 | 0.044 | 0.035 |
|  | 0.000 | 0.000 | 0.032 | 0.092 |
|  |  |  |  |  |
| p05 | 0.124 | 0.112 | -0.082 | -0.046 |
|  | 0.028 | 0.032 | 0.044 | 0.035 |
|  | 0.000 | 0.000 | 0.032 | 0.092 |
|  |  |  |  |  |
| p10 | 0.124 | 0.110 | -0.082 | -0.046 |
|  | 0.028 | 0.031 | 0.044 | 0.035 |
|  | 0.000 | 0.000 | 0.032 | 0.092 |
|  |  |  |  |  |
| p25 | 0.124 | 0.109 | -0.081 | -0.046 |
|  | 0.028 | 0.030 | 0.044 | 0.035 |
|  | 0.000 | 0.000 | 0.031 | 0.091 |
|  |  |  |  |  |
| p50 | 0.121 | 0.105 | -0.079 | -0.046 |
|  | 0.027 | 0.029 | 0.043 | 0.035 |
|  | 0.000 | 0.000 | 0.032 | 0.091 |
|  |  |  |  |  |
| p75 | 0.123 | 0.116 | -0.076 | -0.048 |
|  | 0.028 | 0.031 | 0.042 | 0.036 |
|  | 0.000 | 0.000 | 0.035 | 0.091 |
|  |  |  |  |  |
| p90 | 0.126 | 0.132 | -0.075 | -0.050 |
|  | 0.028 | 0.034 | 0.042 | 0.037 |
|  | 0.000 | 0.000 | 0.035 | 0.092 |
|  |  |  |  |  |
| p95 | 0.126 | 0.133 | -0.075 | -0.050 |
|  | 0.028 | 0.035 | 0.042 | 0.037 |
|  | 0.000 | 0.000 | 0.035 | 0.092 |
|  |  |  |  |  |
| p99 | 0.126 | 0.133 | -0.075 | -0.050 |
|  | 0.028 | 0.035 | 0.042 | 0.037 |
|  | 0.000 | 0.000 | 0.035 | 0.092 |
|  |  |  |  |  |
